# Accumulation of Dietary S‐Methyl Cysteine Sulfoxide in Human Prostate Tissue

**DOI:** 10.1002/mnfr.201900461

**Published:** 2019-09-11

**Authors:** Jack Coode‐Bate, Tharsini Sivapalan, Antonietta Melchini, Shikha Saha, Paul W. Needs, Jack R. Dainty, Jean‐Bapiste Maicha, Gemma Beasy, Maria H. Traka, Robert D. Mills, Richard Y. Ball, Richard F. Mithen

**Affiliations:** ^1^ Quadram Institute Bioscience Colney Lane Norwich NR4 7UQ UK; ^2^ Department of Urology Norfolk and Norwich University Hospitals NHS Foundation Trust Norwich UK; ^3^ Medical School University of East Anglia Norwich UK; ^4^ Norfolk and Waveney Cellular Pathology Service Norfolk and Norwich University Hospitals NHS Foundation Trust Norwich UK; ^5^ The Liggins Institute University of Auckland New Zealand

**Keywords:** broccoli, cancer, glucosinolates, prostate, *S*‐methyl cysteine sulfoxide

## Abstract

**Scope:**

Observational studies have associated consumption of cruciferous vegetables with reduced risk of prostate cancer. This effect has been associated with the degradation products of glucosinolates—thioglycosides that accumulate within crucifers. The possible role of *S*‐methyl cysteine sulfoxide, a metabolite that also accumulates in cruciferous vegetables, and its derivatives, in cancer prevention is relatively unexplored compared to glucosinolate derivatives. The hypothesis that consuming a broccoli soup results in the accumulation of sulfate (a SMCSO derivative) and other broccoli‐derived metabolites in prostate tissue is tested.

**Methods and results:**

Eighteen men scheduled for transperineal prostate biopsy were recruited into a 4‐week parallel single blinded diet supplementation study (NCT02821728). Nine men supplemented their diet with three 300 mL portions of a broccoli soup each week for four weeks prior to surgery. Analyses of prostate biopsy tissues reveal no detectable levels of glucosinolates and derivatives. In contrast, SMCSO is detected in prostate tissues of the participants, with significantly higher levels in tissue of men in the supplementation arm. SMCSO was also found in blood and urine samples from a previous intervention study with the identical broccoli soup.

**Conclusion:**

The consequences of SMCSO accumulation in prostate tissues and its potential role in prevention of prostate cancer remains to be investigated.

## Graphical abstract

**S‐Methyl Cysteine Sulfoxide (SMCSO)** is metabolized by gut bacteria to bioactive products but is also found intact in human plasma urine, and prostate tissue following consumption of a broccoli soup.

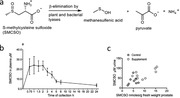

## 1 Introduction

Epidemiological studies have suggested an association between consumption of cruciferous vegetables and prevention of incidence or progression of prostate cancer.^1^, ^2^, ^3^, ^4^ The strength of the association varies greatly between studies, with some studies suggesting consumption of four portions of cruciferous vegetables per week can reduce risk of progression from low‐grade organ‐confined cancer to advanced disease by up to 60%,^1^ while other studies have found no association.^5^

**Figure 1:**
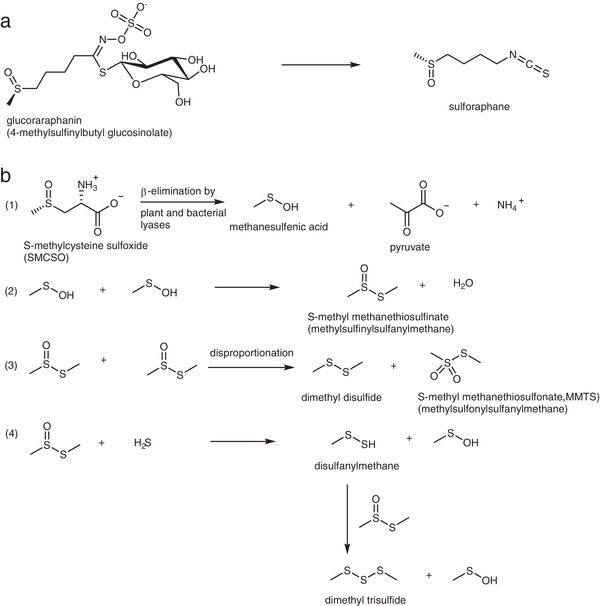
a) 4‐Methylsulphinylbutyl glucosinolate (glucoraphanin) and its hydrolysis to the corresponding isothiocyanate, sulforaphane. b) S‐methylcysteine sulfoxide (SMCSO, methiin) and its degradation products. β‐eliminative enzymatic cleavage of SMCSO produces highly reactive methanesulfenic acid (equation (1)). Spontaneous nucleophilic attack of one molecule of methanesulfenic acid on another leads to the formation of *S*‐methyl methanethiosulfinate (IUPAC name methylsulfinylsulfanylmethane^*^, equation (2)). This can disproportionate (equation (3)) to form dimethyl disulfide and *S*‐methyl methanethiosulfonate (MMTS, IUPAC name methylsulfonylsulfanylmethane^*^). Nucleophilic attack of hydrogen sulfide (a known product of *Brassica* breakdown, though its genesis is unclear) on *S*‐methyl methanethiosulfinate gives disulfanylmethane, which can react in similar fashion with a second molecule of S‐methyl methanethiosulfinate to give dimethyl trisulfide (equation (4)). ^*^Several, often inconsistent names and abbreviations have been used for these compounds.

Cruciferous vegetables accumulate glucosinolates, sulfur‐containing glycosides that are hydrolyzed by either plant or microbial‐derived thioglucosidases to generate an array of biologically active compounds including isothiocyanates and indoles (**Figure** 1a,b).^6^ The biological activities of these derivatives as demonstrated in cell and animal models are widely considered to underpin the protective effects of these vegetables in the human diet.^7^ In addition to glucosinolates, cruciferous vegetables accumulate (+)‐S‐methyl cysteine sulfoxide (SMCSO or methiin),^8^, ^9^ often to significantly higher concentrations (1–4% dry weight) than that of glucosinolates (0.1–0.6%).^10^, ^11^ SMCSO is degraded by plant or microbial cysteine conjugate β lyases to generate a range of biologically active metabolites, including methanic acid, *S*‐methyl methanethiosulfonate, *S*‐methyl methanethiosulfinate, dimethyl disulphide, and dimethyl trisulfide (Figure 1c).^11^, ^12^ Some of these products have been shown to inhibit chemically induced carcinogenesis when fed to rodents,^13^, ^14^, ^15^, ^16^ in a similar manner to that of freeze dried *Brassica*.^17^ The full pathway of SMCSO metabolism in humans, probably initiated by the gut microbiota, remains to be elucidated. It has, however, been shown that inorganic sulfate is a major SMCSO‐derivative in urine.^18^

Despite the reported biological activity of crucifer‐derived metabolites in model systems, it remains challenging to provide a satisfactory mechanistic explanation of how consuming relatively few portions of cruciferous vegetables per week can lead to a reduction in risk of aggressive prostate cancer. Several pharmacokinetic studies have shown that the concentration of glucosinolates and isothiocyanates such as sulforaphane in plasma following a portion of cruciferous vegetables is transient, and significantly lower (*C*_max_ < 1 µm) than that frequently used in model systems (typical exposure of 5–20 µm for 24 h).^19^, ^20^ One explanation may be that there is accumulation of the plant‐derived compounds and/or their biological‐active derivatives in prostate tissue, as has been shown for lycopene derived from tomatoes,^21^ resulting in localized exposure of tissue to sufficiently high concentrations to induce changes that may result in reduction in carcinogenesis and growth of cancerous clones.

In this study, we investigate whether consuming a broccoli soup rich in 4‐methylsulphinybutyl glucosinolate (“glucoraphanin”), the precursor of the isothiocyanate sulforaphane, and SMCSO can result in the accumulation of sulfate, the reported major derivative from SMCSO,^18^ and other broccoli‐derived metabolites in human prostate tissue. As we unexpectedly found un‐metabolized SMCSO in prostate and adipose tissues, we additionally quantified SMCSO in tissue samples from prostate from men who had not been involved in a dietary study, and report the level of SMSCO in plasma and urine of volunteers over a 24 h period following consumption of a single portion of the broccoli soup as part of a previously described dietary intervention study^19^ to provide further insight to the bioavailability and metabolism of SMCSO.

## 2 Experimental Section

### 2.1 SAP Study Design

**Figure 2:**
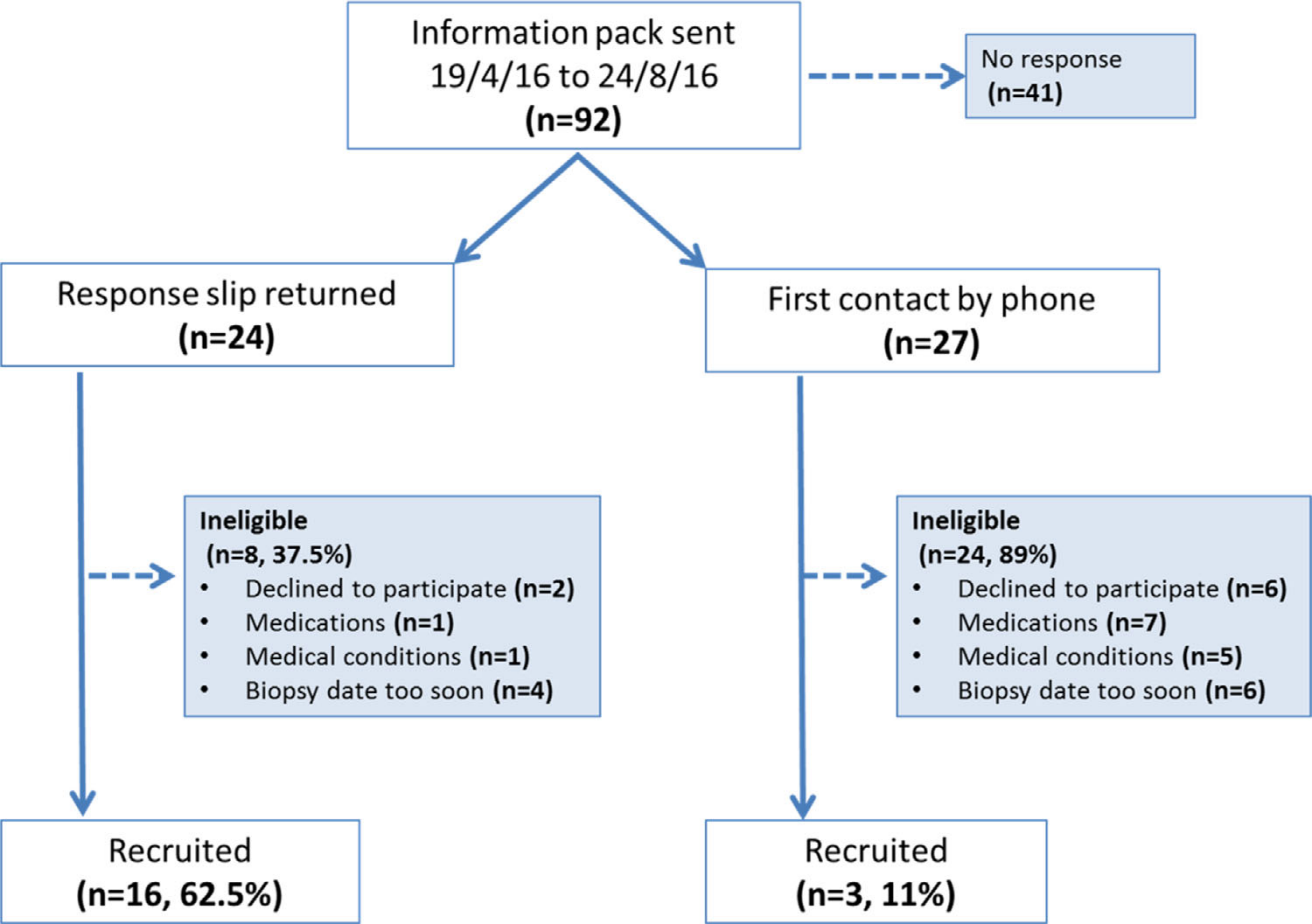
Flow chart of pathways to recruitment.

The SAP study (**Figure** 2) was a two‐arm, parallel un‐blinded, dietary‐supplementation study, delivering a short, high‐dose broccoli soup supplement to a habitual diet to men in the pre‐biopsy window before a trans‐perineal prostate biopsy (TPB). The primary aim was to determine whether supplementation of a diet with a broccoli soup would enhance the levels of sulfate and other broccoli‐derived metabolites in the prostate of men scheduled for prostate biopsy. Eighteen men were randomized to either continue their normal diet (non‐supplementation controls) or receive three portions of a glucoraphanin‐rich broccoli soup (300 g) per week for a minimum of 4 weeks manufactured from a broccoli cultivar heterozygous for a Myb28^villosa^ allele, as previously described.^19^ On the study day, samples of whole blood, urine (following a digital rectal prostatic massage), prostate, and peri‐prostatic adipose tissue were collected. The protocol was approved by the Human Research Governance Committee (HRGC IFR 01/2016) at Quadram Institute Bioscience and given full ethical approval by The East of England – Cambridge East Research Ethics Committee (ref: 16/EE/0054). The trial was registered on a publicly accessible database (ClinicalTrials.gov, NCT02821728).

The sample size for the SAP study was calculated based upon unpublished data of presence of sulfate within biopsy tissue of men consuming broccoli in which men randomized to a 12‐month broccoli intervention had shown a significant accumulation of sulfate in prostate tissue after the intervention period compared to baseline. To detect a difference of 1.735 (arbitrary units) at a 5% significance level with 90% power and assuming a within‐group SD of 1.056 required a sample size of nine individuals in each group (total 18). These sample sizes were calculated for a two‐group study design (broccoli supplementation vs no supplementation) assuming a two‐sided comparison (i.e., to detect a difference rather than a higher level).

### 2.2 Dosage Information

**Figure 3:**
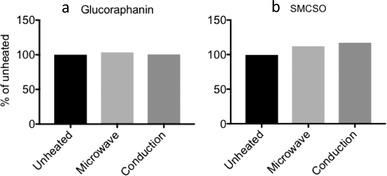
The effect of heating soup to boiling on amount of a) glucoraphanin and b) SMCSO compared to unheated control by microwave or conduction heating. Columns are means of two independent samples.

One portion of soup contained 280 ± 8.8 µmoles of glucoraphanin and 1513 ± 36.8 µmoles of SMCSO. The soup was provided frozen and heated up prior to consumption using a standard procedure. Heating either by microwave or conduction had no effect on SMCSO or glucoraphanin concentration (**Figure** 3).

### 2.3 Study Population

Men who were on the waiting list for TPB at Norfolk and Norwich University Hospital as part of their routine clinical care were targeted for enrolment if they were between the ages of 18 and 80 years and had a body mass index of between 19.5 and 35 kg m^–2^. The inclusion criteria included those with either a previous negative TRUS‐guided prostate biopsy or those with a histologically confirmed abnormality, including diagnosis of prostate cancer that required further investigation or surveillance. Exclusion criteria included those taking 5α reductase inhibitors, testosterone replacement medicines, warfarin, and dietary supplements, and those with a diagnosis of diabetes. Eligible patients were provided with an information pack concerning the rationale for the study and details of the supplementation. Those who wished to take part were given a 3‐day period of reflection before providing formal written consent. Randomization was performed by a third party with use of an online randomization generator (http://www.randomization.com).

### 2.4 Study Day Procedure

A sample of whole blood for genotyping was obtained via the intravenous cannula inserted for use by the anesthetist, thus avoiding additional venipuncture. After prostatic massage, a first‐pass urine sample was collected. TPB was performed under general or spinal anesthesia with appropriate antibiotic prophylaxis. Once anesthetized, the patient was positioned supine with their legs elevated in stirrups. A template grid was placed on the skin of the perineum and the prostate visualized by trans‐rectal ultrasound scan. After measuring the volume of the prostate, the whole gland was systematically sampled through the template grid, which had holes spaced at 5 mm intervals. For the SAP study, eight biopsy cores of prostate tissue were taken from a region of the prostate not known or suspected to contain cancer. Five of these were snap frozen for metabolite analyses, two in RNAlater, and one in methanol. Two additional cores were taken from peri‐prostatic adipose tissue and snap frozen.

### 2.5 Cruciferous Vegetable Food Frequency Questionnaire

To evaluate the habitual intake of cruciferous vegetables of all volunteers, a cruciferous vegetable food frequency questionnaire developed and validated by the University of Arizona^22^ was completed during the study day.

### 2.6 Sulfate and SMCSO Extraction from Prostate Tissue

Snap‐frozen tissue cores were individually weighed on a high‐sensitivity balance and transferred to screw‐top tubes. Two hundred microliters of cold Milli‐ Q^®^ water and 300 µg of autoclaved, acid‐washed 710 to 1180 µm glass beads were added to each tube. The tissue was completely homogenized using a DNA Fast‐Prep^®^ (MP Biomedicals) at 4.0 m s^–1^ for three cycles of 60 s each. The samples were then placed on a revolving shaker for 15 min at 4^o^ C. The tubes were centrifuged at 17 000 × *g* for 10 min at 4 ^o^C and 50 µL of supernatant transferred to a new Eppendorf. 10 µL of 50% trichloroacetic acid (TCA) were added to each sample to precipitate proteins. The centrifugation step was repeated and 50 µL of supernatant transferred to HPLC insert vials for analysis by LC–MS/MS.

### 2.7 Sulfate and SMCSO Extraction from Urine

Aliquots of urine were thawed on ice, vortexed briefly and 100 µL transferred to an Eppendorf tube. A tenfold dilution in 5% TCA was performed, the sample vortexed and incubated on ice for 10 min to precipitate proteins. The sample was centrifuged at 14 000 × *g* for 10 min at 4 ^o^C and the supernatant transferred to an HPLC vial for analysis by LC–MS/MS.

### 2.8 Quantification of Sulfate and SMCSO by LC–MS/MS

Stock solutions of 1 mg mL^–1^ sulfate (Sigma) and SMCSO (LKT laboratories Inc.) were generated by dilution of weighed powder into Milli‐Q water. Serial fivefold dilutions were performed in the relevant matrix to produce 6‐point calibration curves from 10 to 0 µg mL^–1^ on the day of analysis. Sulfate was quantified using an Agilent 6490 triple‐quad LC–MS mass spectrometer (Agilent technologies) with Thermo Scientific Hypercarb, Porous Graphitic Carbon (PGC) (3 × 50 mm, 3 µm) column. Two microliters were injected from each sample, with separation by 1% formic acid in Milli‐Q water (mobile phase A) and 1% formic acid in methanol (mobile phase B). The gradient started at 8% mobile phase B, increasing to 70% over 5.5 min and returned to 8% mobile phase B for re‐equilibration over the last 9.5 min. The column temperature was maintained at 60 °C and the flow rate at 0.3 mL min^–1^. The LC eluent flow was sprayed into the mass spectrometer interface without splitting. Inorganic sulfate was monitored using MS in MRM mode (*m*/*z* = 97/80 transition) with ESI in the negative polarity. The MS source parameters were: gas temperature 200 °C, gas flow 16 L min^–1^, sheath gas temperature 400 °C, sheath gas flow 12 L min^–1^, capillary voltage 3000 V and nozzle voltage 1000 V. Quantification of sulfate was performed by peak area against the matrix‐matched standard curve, and identification by retention time and product ions.

SMCSO was quantified with the Agilent system described above. Samples were injected at 2 µL eluted at a flow rate of 0.3 mL min^–1^ on an Agilent SB‐AQ 1.8 µm (100 × 21 mm) C18 column. Separation was carried out using 10 mm ammonium acetate + 0.05% hetafluorobutyric acid in Milli‐Q water (mobile phase A) and 10 mm ammonium acetate + 0.05% hetafluorobutyric acid in 90% methanol (mobile phase B). The gradient started at 2% mobile phase B, increasing over 2 min to 5% B and returning to 2% mobile phase B for re‐equilibration over the last 2 min. The column was set at 20 °C due to the instability of SMCSO at high temperatures. The LC eluent flow was sprayed into the mass spectrometer interface without splitting. SMCSO ion was monitored by MS in MRM mode (*m*/*z* = 87.9) in positive polarity with ESI. The source parameters were: gas temperature 200 °C, gas flow 16 L min^–1^, sheath gas temperature 300 °C with a sheath gas flow of 11 L min^–1^, a nebulizer pressure of 50 psi, and capillary voltage 3500 V. Quantification was performed by peak area against the matrix‐matched standard curve, and identification by retention time and product ions.

### 2.9 Glucoraphanin and Glucoerucin Extraction from Tissue and UPLC–MS/MS Analysis

Snap‐frozen tissue cores were individually weighed on a high‐sensitivity balance and transferred to screw‐top tubes. Two hundred microliters of cold Milli‐Q water and 300 µg of autoclaved, acid‐washed 710 to 1180 µm glass beads (Sigma) were added to each tube. The tissue was completely homogenized using a DNA Fast‐Prep (MP Biomedicals) at 4.0 m s^–1^ for three cycles of 60 s each. The samples were then placed on a revolving shaker for 15 min at 4 ^o^C. The tubes were centrifuged at 17 000 × *g* for 10 min at 4 ^o^C and 50 µL of supernatant transferred to a new Eppendorf. Ten microliters of 50% trichloroacetic acid (TCA) were added to each sample to precipitate proteins. The centrifugation step was repeated and 50 µL of supernatant transferred to HPLC insert vials for analysis by UPLC–MS/MS. GR and GE were separated with 0.2% formic acid in water (mobile phase A) and 0.2% formic acid in acetonitrile (mobile phase B) using a Kinetex 1.7 µm XB‐C18 100 Å 100 × 2.1 mm UPLC column. The gradient started at 5% mobile phase B increasing over 7 min to 80% mobile phase B and finally re‐equilibrated to 5% mobile phase B for 2 min. The LC eluent flow was sprayed into the mass spectrometer interface without splitting. GR ion and GE ion were monitored using MS in MRM mode (GR 436/97; GE 412/97) in negative polarity with ESI.

### 2.10 Analyses of Glucoraphanin, Sulforaphane, and Sulforaphane Conjugates by LC–MS/MS

The analyses of sulforaphane and sulforaphane conjugates in prostate tissue, plasma, and urine was as previously described.^19^

### 2.11 Analyses of SMCSO from Prostatectomy Tissue Samples

To further explore the presence of un‐metabolized SMCSO in the prostate gland of men who had not been within a dietary study, tissue samples were obtained from patients who had endoscopic extraperitoneal radical prostatectomies. Access to tissue and subsequent analyses were undertaken under ethical approval granted from the Faculty of Medicine and Health Sciences Research Ethics Committee of the University of East Anglia (FMHS 20122014–37). The prostate gland was removed from the abdominal cavity immediately after resection and rapidly biopsied after extraction to avoid ischemic artifacts using a standard core biopsy instrument, as previously described.^23^ The extracted whole prostatectomy specimen was placed on a surgical table, the apex and base of the gland were identified, and the prostate cut transversely (axial section) half way along the gland. With the use of the midline and the urethra as guides, biopsies were taken from the peripheral zones avoiding obvious tumor sites where feasible. Two cores were taken from each of four glands for analyses. The tissue was snap frozen and analyzed for the presence of SMCSO as described above.

### 2.12 Bioavailability and Excretion of SMCSO

To quantify the amount of SMCSO in plasma and urine from a single portion of soup, samples were analyzed from a previously described acute intervention study (NCT02300324).^24^ In this study, ten volunteers consumed a single portion of the identical soup to that used in the SAP study as described above as part of a three‐phase cross‐over study. Briefly, men and woman aged 18–65 years with a BMI between 19.5 and 35 kg m^–2^ were enrolled into a dietary intervention trial undertaken at the Human Nutrition Unit of the Quadram Institute Bioscience Subjects were recruited on the basis of fasted (≥8 h) screening blood/urine samples and a completed health questionnaire. The study involved a 48‐h pre‐intervention diet restriction, a study day involving a 9‐h stay at the HNU and a sample collection the following morning (24 h post soup consumption) followed by a washout period of 2 weeks. Participants (*n* = 10) were required to follow a glucosinolate‐free diet as well as avoiding alcohol, spicy food and *Allium* for 48 h prior to each study day until the collection of the 24‐h sample for each phase. This dietary restriction was required to ensure that glucosinolates and SMCSO from other food sources did not have an impact on the study results. During each study day, eleven blood samples (10 mL) were collected after the consumption of the soups at the following timepoints: 0, 30, 45, 60, 90, 120, 180, 240, 360, and 480 min, and 24 h. Six urine samples were collected at the following timepoints: 0, 0–2, 2–4, 4–6, 6–8, and 8–24 h. Ethical approval was obtained from the Human Research Governance Committee at QIB(IFR06/2014) and the National Research Ethics Service East of England Norfolk Ethics Committee (reference 14/EE/1121). The study was registered on clinictrials.gov (NCT02300324).

### 2.13 SMCSO and Glucosinolates in Cruciferous and Alliaceous Vegetables

SMCSO and glucosinolates was quantified in a selection of cruciferous and alliaceous vegetables purchased from a retail outlet to provide an indication of the relative amounts of these metabolites that occur in these vegetables.

### 2.14 Data Availability

The datasets generated and analyzed during the current study are available from the corresponding author upon request.

## 3 Results

### 3.1 Recruitment

Ninety‐two information packs were sent to patients on the waiting list for TPB, 24 potential participants returned response letters, and a further 27 were contacted by telephone. The conversion rate to full participation was high (62.5%) amongst those who returned the response slip, primarily limited by a scheduled biopsy date that was too soon for the supplementation. Those that were first contacted by telephone were less likely to meet the inclusion criteria and only three (11%) subsequently decided to enrol on the study. One volunteer in the non‐supplementation arm dropped out from further participation due to illness on the study day (Figure 2).

### 3.2 Participant Demographics

Block randomization distributed participants equally to both study arms, with no statistically significant difference (unpaired *t*‐test) in age, BMI, prostate specific antigen (PSA), or PSA density (**Table** 1). The mean BMI for men in both arms fell into the overweight category, and as anticipated for men recommended to undergo TPB the PSA in both groups was above the age‐adjusted normal range.

**Table 1:** Participants. There are no differences between the two arms of the study

	Control	Supplementation
Age years	64.7 ± 5.39^a^	68.6 ± 6.46
BMI [kg m^–2^]	26.8 ± 3.29	28.1 ± 2.58
PSA [ng ml^–1^]	7.8 ± 4.17	8.7 ± 2.64
PSA density [ng mL cm^–3^]	0.14 ± 0.101	0.12 ± 0.05

a Mean ± SD.

### 3.3 Participant Cancer Grade and Volume

Despite clinical suspicion of prostate cancer indicating the need for TPB, histology reports revealed 11 of the 18 participants had benign diagnoses. There were no significant differences in the overall distribution of cancer diagnoses between the two groups. Study biopsies were taken from the right anterior quadrant of the prostate or the most remote region from any focal areas of clinical suspicion. Three volunteers were diagnosed with cancer in the quadrant sampled (two in the supplementation group and one in the non‐supplementation group). However, the volume of tissue affected was small (<15%).

### 3.4 Cruciferous Vegetable Intake

One volunteer failed to fully complete and return the Arizona cruciferous vegetable food frequency questionnaire. As part of their habitual diet, the supplementation group and non‐supplementation group consumed similar quantity of cruciferous vegetables per day (67 ± 52.9 g and 72 ± 54.5 g, respectively).

### 3.5 Dietary Supplementation Resulted in Accumulation of SMCSO in Prostate Tissue

**Figure 4:**
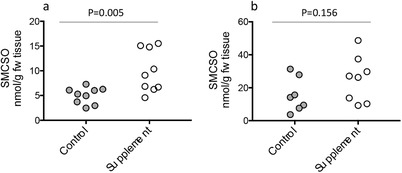
a) SMCSO in prostate and b) SMCSO in peri‐prostatic tissue in control and supplement groups. *p*‐Values are from Student's *t*‐tests.

**Figure 5:**
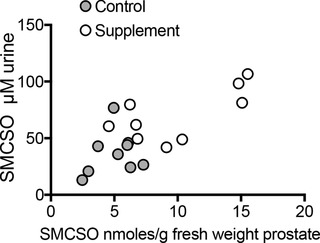
SMCSO is prostate and urine.

**Figure 6:**
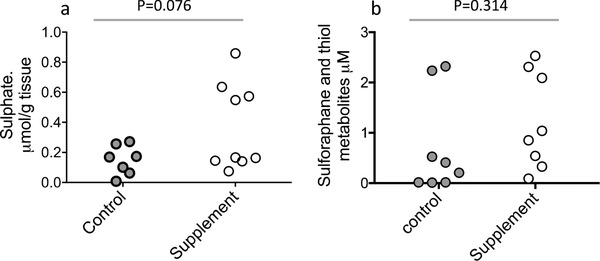
a) Sulfate in prostate tissue of control and supplement groups. b) Sulforaphane in urine of control and supplement groups. *p*‐Values are from Student's *t*‐tests.

Higher levels of SMCSO were found in prostate tissue samples following the broccoli supplementation compared to the non‐supplementation arm (**Figure** 4a). SMCSO was also detected in peri‐prostatic adipose tissues at higher levels than in prostate tissue but without significant difference between the two groups (Figure 4b). SMCSO was higher in urine in the supplement group compared to the control (*p* = 0.004, Student's *t*‐test) and correlated with the levels in prostate (**Figure** 5, *r* ^2^ = 0.52, *p* = 0.0007, linear regression). There was no significant difference in the level of sulfate in prostate tissue between the two dietary arms (**Figure** 6a), and there was no correlation between prostate sulfate and prostate SMCSO (*p* = 0.29, linear regression).

No glucoraphanin or sulforaphane (and its thiol conjugates) was detected in prostate tissue. Sulforaphane was detected in urine from participants in both arms of the study (Figure 6b). As would be expected, levels of sulforaphane were lower in men in the control arm who were not provided with the broccoli soup. Two men in this arm had relatively high levels of sulforaphane and this was a result of the consumption of broccoli within the previous 24 h, as evident through the Arizona cruciferous vegetable food frequency questionnaire.

### 3.6 SMCSO is Present in Tissue from Radical Prostatectomies of Men Who Had Not Been Associated with Dietary Studies

**Figure 7:**
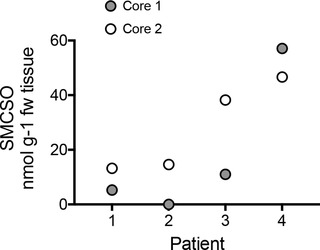
SMCSO in two cores from four patients who had undergone radical prostatectomies.

SMCSO was detected in seven of the eight tissue samples obtained from radical prostatectomies (**Figure** 7). Levels in patient four exceeded those that occurred within the supplementation study.

### 3.7 SMCSO is Found in Plasma Following Consumption of Broccoli Soup

**Figure 8:**
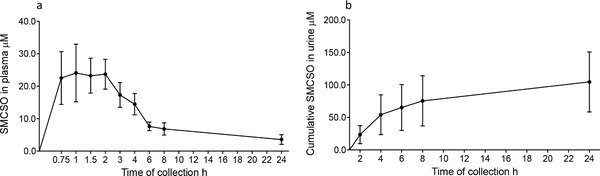
a) SMCSO in plasma and b) in urine following consumption of single portion of broccoli soup at time 0. Points represent mean ± SD.

When samples from a previous dietary intervention study were analyzed, SMCSO was detected in plasma and urine from samples collected at intervals within 24 h following consumption of a single portion of the same broccoli soup as in the SAP study (**Figure** 8). Peak plasma concentration occurred after 1.4 h and peaked at 198 µmol L^−1^. After 24 h, 6% of consumed SMCSO has been excreted in urine (**Table** 2).

**Table 2:** Pharmacokinetic parameters of glucoraphanin, sulforaphane + metabolites and SMCSO following consumption of 300 mL portion of broccoli soup. Data for glucoraphanin and sulforaphane has previously been published by Sivapalan et al.^19^

	Glucoraphanin	Sulforaphane and metabolites^a^	SMCSO
Amount consumed [µmoles]	280 ± 8.8	0	1513 ± 36.8
Plasma			
AUC [µmol h L^−1^]	0.15 ± 0.08	4.92 ± 3.77	195.34 ± 40.42
C_max_ [µmol L^−1^]	0.03 ± 0.01	0.37 ± 0.26	28.03 ± 5.39
T_max_ [h]	2.23 ± 0.09	9.20 ± 5.27	1.70 ± 0.35
C_24h_ [µmol L^−1^]	<0.01	0.07 ± 0.06	4.66 ± 1.52
Urine			
Total excreted in 24 h [µmoles]	1.44 ± 0.66	23.14 ± 16.17	104.71 ± 46.16
Percentage excreted after 24 h	0.51 ± 0.24	8.26 ± 5.78^b^	6.92 ± 2.33

a Sum of sulforaphane, sulforaphane‐N‐acetyl cysteine; sulforaphane‐glutathione; sulforaphane‐cysteine; sulforaphane‐cystine‐glycine.

b As a percentage of glucoraphanin

### 3.8 SMCSO and Glucosinolates in Cruciferous and Alliaceous Vegetables

**Figure 9:**
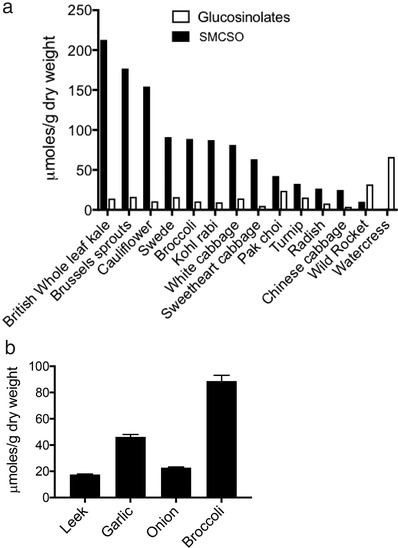
a) The concentration of SMCSO and glucosinolates in samples of cruciferous vegetables. Each bar represents level in pooled sample of vegetables purchased from retail outlet. The precise cultivars are unknown. b) Mean ± SD of three samples of leek, garlic, onion, and broccoli purchased from retail outlet. The cultivars are unknown.

A preliminary survey of SMCSO in cruciferous and alliaceous vegetables indicated that SMCSO occurs at higher concentrations than glucosinolates in all *Brassica* vegetables, but is low in rocket (*Diplotaxis tenufolia*) and watercress (*Nasturtium officinale*; **Figure** 9a). Furthermore, SMCSO occurs at higher concentrations in broccoli than in leek, garlic, and onion (Figure 9b).

## 4 Discussion

Diets rich in cruciferous vegetables have been associated with reduced risk of cancer at several sites, including prostate,^1^, ^2^ breast,^25^ lung,^26^, ^27^ and colon,^28^ and with reduce risk of cardiovascular disease and atherosclerosis.^29^, ^30^, ^31^ It is widely assumed that these protective effects are at least partially mediated by glucosinolate degradation products. Indeed, a study recently demonstrated that consuming a high‐glucoraphanin broccoli weekly for 1 year can successfully suppress transcription of predominantly oncogenic pathways in prostate tissue of men with localized prostate cancer.^32^ However, the possible contribution of SMCSO and its derivatives have received scarce attention. In this study, we report that consuming three portions of a glucoraphanin‐rich soup per week for 4 weeks did not result in accumulation of glucoraphanin or sulforaphane in prostate tissue. Sulforaphane was detected in the urine of volunteers in both the control and supplementation arms of the study (Figure 7). As has previously been described,^19^ the level of sulforaphane in the urine reflects the consumption of glucoraphanin containing vegetables within the previous 24 h, as opposed to being a longer term marker of chronic consumption. In contrast to sulforaphane and other isothiocyanates, SMCSO and its derivatives accumulate and are excreted over a longer period of time and may provide a better biomarker of cruciferous vegetable consumption, as has previously been suggested,^33^ but would also reflect consumption of alliaceous vegetables.

A previous study has reported that an oral gavage of 5 µmoles (110 µmoles kg^–1^ body mass) of sulforaphane in mice resulted in a transient presence of sulforaphane in prostate of between 50 and 100 pmoles after 6 h.^34^ If we assume a 8% conversion of glucoraphanin to sulforaphane,^19^ the human dose equivalent of glucoraphanin would be 8.8 mmoles or 32 portions of broccoli soup based upon the US Food and Drug Administration algorithm for calculating human dose equivalents from mouse studies.^35^

In contrast, to the lack of sulforaphane, we found SMCSO to be present in the prostate and peri‐prostatic tissue of all participants, and at significantly greater levels in the supplementation arm than the non‐supplementation arm (Figure 4a). SMCSO also occurred in urine and were correlated with that in prostate tissue (Figure 4b). The presence of SMCSO in both prostate and adipose tissues of all volunteers (control and supplemented) was unexpected. It is likely that it is of dietary origin as there are no known routes by which mammals can synthesize SMCSO.^9^ Furthermore, we found SMCSO in prostate tissue of men who had not been part of a dietary study (Figure 7).

Through the analyses of plasma and urine from volunteers who consumed a single portion of the broccoli soup, we found un‐metabolized SMCSO occurring in plasma with C_max_ 1.5 h after consumption (Figure 6) suggesting rapid absorption in the upper GI tract and recovered about 6% of ingested SMCSO as un‐metabolized SMCSO in urine after 24 h. The plasma concentration of SMCSO was about 1000‐fold higher than that of glucoraphanin or sulforaphane (Table 2).

Previously, there has been only a single study of the metabolism of SMCSO in humans. Waring and colleagues describe the feeding of ^35^S‐labeled SMCSO to human volunteers, and the recovery of 96% of the label in urine after 14 days, of which 40% was inorganic sulfate, indicating almost complete absorption of SMCSO (and/or its colonic microbiological derivatives).^18^ How SMCSO was metabolized is unknown, but it is likely that it was due to initial cysteine‐S‐conjugate β lyase activity by gut microbiome, as opposed to mammalian enzyme activity. Together with the study of Waring and colleagues,^18^ this suggests that about 90% of SMCSO that is consumed is metabolized in vivo.

The pharmacokinetics of glucoraphanin and sulforaphane contrast with that of SMCSO. Very little of ingested glucoraphanin is absorbed intact. Between 2% and 15% is recovered in urine as sulforaphane and associated thiol conjugates after 24 h, with little or no subsequent excretion.^19^ It is assumed that the unaccounted glucoraphanin is voided with feces. In contrast, the majority (97%) of SMCSO is absorbed, either as un‐metabolized SMCSO or as unknown derivatives, and excretion of SMCSO (and its derivatives) occurs over several days.^18^ The paucity of studies on human metabolism and pharmacokinetics of SMCSO hinders the interpretation of the current study. It was apparent that there was considerable variation in the levels of SMCSO in prostate and peri‐prostatic adipose tissues within both arms of the experimental study and within tissue obtained from men not on the study. It is conceivable that this is due to different dietary habits of the tissue donors with respect of consumption of cruciferous and alliaceous vegetables. Further more detailed and longer‐term dietary intervention studies are required to explore the basis to this observed variation in SMCSO content of human tissues.

The major interest in the metabolism of SMCSO has been as the causal agent of hemolytic anemia in cattle or sheep. When fed cruciferous fodder crops SMCSO is cleaved within the rumen due to microbial cysteine‐S‐conjugate β lyase activity to generate the highly reactive methanesulfenic acid that reacts with itself to produce an array of products (Figure 1), including MMTS, dimethyl disufide, and dimethyl trisulfide.^9^, ^12^ Within the blood, these products are thought to induce oxidative stress through depletion of reduced glutathione leading, among other metabolic effects, to Heinze body formation and hemolysis,^36^ although SMCSO has also been speculated to have a positive effect on human health.^36^ Subsequent studies should seek to quantify SMCSO catabolic products in the circulation and their accumulation in tissues. This will require a greater understanding of the catabolism of SMCSO and development of analytical methods. Several SMCSO catabolic products, such as sulphate, may be similar or identical to those generated by endogenous human metabolism.

The presence of un‐metabolized SMCSO in prostate tissue may result from delivery from the plasma or urinary reflux. While we have demonstrated the presence of SMCSO in prostate tissue, it is not clear whether it would have any metabolic activity itself, or whether any activity would be of a consequence of its degradation to biologically active derivatives. It may be noteworthy that there is increasing evidence for a microbiological community associated both with the urinary tract and the prostate itself that may metabolise SMCSO to its active derivatives.^37^, ^38^, ^39^

Despite the occurrence of SMCSO at higher concentrations than glucosinolates in *Brassica* vegetables (Figure 9a), this compound has received little attentions regarding its potential contribution to the health benefits attributed to cruciferous vegetable consumption. This may be due to the complexities of its catabolism and distinguishing its metabolites from those of endogenous human metabolism. SMCSO also occurs in alliaceous vegetables such as onions and garlic (Figure 9b), along with additional *S*‐alk(en)yl cysteine sulfoxides.^40^ Consumption of *Allium* has also been associated with reduction in prostate cancer,^41^ which may be mediated by these metabolites and their derivatives. It is likely that *S*‐alk(en)yl cysteine sulfoxides such as alliin from garlic may also accumulate in tissues following consumption in a similar manner to SMCSO.

This study highlights the potential role of SMCSO in mediating the putative effects of diets rich in cruciferous and alliaceous vegetable consumption in prevention of aggressive prostate cancer, and possibly other health benefits. In contrast to glucosinolates, almost 100% of SMCSO appears to be absorbed from the GI tract with the majority being metabolised probably by the gut microbiome to biologically active derivatives. Un‐metabolized SMCSO is found in plasma and appears to accumulate in prostate. Further studies are required to determine if this is of biological importance. The relative importance, if any, of sulforaphane and other glucosinolate derivatives, and SMCSO and its catabolic products to the health benefits of diets rich in cruciferous vegetables requires further investigation. Future studies need to focus on gaining a greater understanding of the microbial and human metabolism of SMCSO, the analyses of SMCSO catabolic products in the circulation and their accumulation in peripheral tissues, and the biological activity of SMCSO and its catabolic products.

Finally, it has been reported that the human prostate does have a commensal bacterial community.^37^, ^42^ It is therefore possible that SMCSO accumulates in prostate tissue, and then is catabolized by a resident microbial population to bioactive components that may inhibit the proliferation of cancerous cell lines.

## Conflict of Interest

The authors declare the following competing interests: R.F.M., M.H.T. and A.M. are co‐inventors in two patents (PCT/GB2017/050838, GB1605013.0) that cover combinations of a composition comprising glucoraphanin and SMCSO for the treatment or prevention of prostate cancer. The other authors declare no conflict of interest.
